# Assessing feasibility and acceptability of home-care based rehabilitation from the perspective of stroke patients and their informal caregivers

**Published:** 2012-09-04

**Authors:** M Viana, Silvina Santana

**Affiliations:** Institute of Electronics Engineering and Telematics of Aveiro, Department of Economics, Management and Industrial Engineering, University of Aveiro, Aveiro, Portugal; Associate Professor with Aggregation, Department of Economics, Management and Industrial Engineering; Institute of Electronics Engineering and Telematics of Aveiro, and Research Unit in Governance, Competitiveness and Public Policies, University of Aveiro, Aveiro, Portugal

**Keywords:** home-care rehabilitation, feasibility, acceptability, patient perceptions, informal caregiver’s perceptions, stroke, Portugal

## Abstract

**Purpose:**

To report on stroke patients and their informal caregivers’ perceptions and experiences regarding feasibility and acceptability of home-care based rehabilitation, in the context of the implementation of an EHSD procedure.

**Theory:**

EHSD procedures are considered promising alternatives to conventional care in stroke rehabilitation, as they seem to be able to reduce costs with illness and to accrue important benefits to patients and their informal caregivers. The literature in this area provides some guidelines of how to organize and assess this type of intervention, regarding the impact on patient’s functionality and related costs. Less investigated are the perspectives of patients and their informal caregivers, namely, their perceptions and experiences regarding feasibility and acceptability of home-care based rehabilitation.

**Methods:**

Data were collected using focus group discussion. We conducted two focus groups with key-informants, selected patients and caregivers, chosen due to the diversity and deepness of their cases, aiming at improving both evidence-based knowledge and theoretical framework. A group of three researchers with specific roles joined each focus group, namely a moderator, a recorder and a coordinator. During the sessions, participants were encouraged to speak until all views were expressed. The data were recorded, transcribed and analyzed. The content analysis was performed with NVivo 9 which allowed us to identify the main themes of each topic under discussion.

**Results and conclusions:**

Home-care based rehabilitation seems to be feasible and acceptable for patients and their caregivers. Our results point out the advantages of home-care over traditional models. Moreover, patients and their caregivers identified aspects that can be improved in home-based services.

Powerpoint presentation available at http://www.integratedcare.org at congresses – San Marino – programme.

